# Nanoemulsions as Edible Coatings: A Potential Strategy for Fresh Fruits and Vegetables Preservation

**DOI:** 10.3390/foods10102438

**Published:** 2021-10-14

**Authors:** Josemar Gonçalves de Oliveira Filho, Marcela Miranda, Marcos David Ferreira, Anne Plotto

**Affiliations:** 1 School of Pharmaceutical Sciences, São Paulo State University (UNESP), Rodovia Araraquara—Jaú Km 1, Araraquara 14800-903, SP, Brazil; josemar.gooliver@gmail.com (J.G.d.O.F.); mmiranda.bio@gmail.com (M.M.); 2 Embrapa Instrumentação, Rua XV de Novembro, 1452, São Carlos 13560-970, SP, Brazil; 3 ARS Horticultural Research Laboratory, United States Department of Agriculture, 2001 South Rock Road, Fort Pierce, FL 34945, USA

**Keywords:** nanotechnology, wax coating, natural antimicrobials, essential oils, nanocoatings, post-harvest, bioactive compounds, quality, preservation methods, nanomaterials

## Abstract

Fresh fruits and vegetables are perishable commodities requiring technologies to extend their postharvest shelf life. Edible coatings have been used as a strategy to preserve fresh fruits and vegetables in addition to cold storage and/or controlled atmosphere. In recent years, nanotechnology has emerged as a new strategy for improving coating properties. Coatings based on plant-source nanoemulsions in general have a better water barrier, and better mechanical, optical, and microstructural properties in comparison with coatings based on conventional emulsions. When antimicrobial and antioxidant compounds are incorporated into the coatings, nanocoatings enable the gradual and controlled release of those compounds over the food storage period better than conventional emulsions, hence increasing their bioactivity, extending shelf life, and improving nutritional produce quality. The main goal of this review is to update the available information on the use of nanoemulsions as coatings for preserving fresh fruits and vegetables, pointing to a prospective view and future applications.

## 1. Introduction

Fruits and vegetables are important sources of minerals, vitamins, and fibers, which are essential for human’s well-being, and their consumption has been associated with several beneficial effects on human health. The demand for those benefits has considerably increased over the years due to consumer preference for natural products and changes in lifestyle [1]. In this sense, fruits and vegetables are an important component of the human diet.

After they are harvested, fruits and vegetables continue the respiration process, consuming O_2_ and releasing CO_2_ and water. Consequently, lipids, proteins, organic acids, and carbohydrates are metabolized and energy replacement is compromised, as the vegetable or fruit is separated from the mother plant [2]. Over time, quality characteristics such as color, flavor, weight, nutritional value, and bioactive compounds continue to deteriorate as a result of senescence [3]. The water released during the respiration process plays an important role in the postharvest quality of fresh fruits and vegetables and can result in loss of nutritional value, soft texture, sagging, wrinkling, and withering [4].

Although waxes were used to preserve citrus fruit in ancient China, it was not until the twentieth century that edible coatings based on emulsions were developed to preserve the quality of fresh fruits and vegetables [5]. These emulsions are typically formulated from oils (vegetable- or animal-derived), waxes (paraffin, carnauba wax, candelilla, or beeswax), and resins (shellac or wood rosin). Furthermore, polymer-based coating solutions can have additional functionality when formulated with plant essential oils having antimicrobial activity [6]. Due to their hydrophobic nature, oils and waxes have proven to be an efficient technology for fruits and vegetables preservation post-harvest, as they are able to minimize water loss and gas exchange and improve and/or preserve the physicochemical properties, such as color, firmness, fresh appearance, and microbial protection [7,8,9,10].

Recently, nanotechnology was introduced as a new tool for making coatings based on emulsions with improved properties and functionalities. Coatings are made of macro- or microemulsions (conventional) or nanoemulsions, for which the latter can be considered a conventional emulsion with very small particles. Droplets in nanoemulsions are on a nanoscale (particle radius less than 100 nm) dispersed in an aqueous solution [11]. This changes the physical properties of the coating by further reducing moisture migration, gas exchange, oxidative reactions, and suppressing pathogenic growth (microorganisms), product deterioration and enhancing control of physiological disorders [12]. In addition, coatings based on nanoemulsions have shown to be promising vehicles for several active compounds, such as oil-soluble vitamins, antimicrobials, flavors, and nutraceuticals, which may further contribute to maintenance of food product quality attributes [13]. 

**Figure 1:**
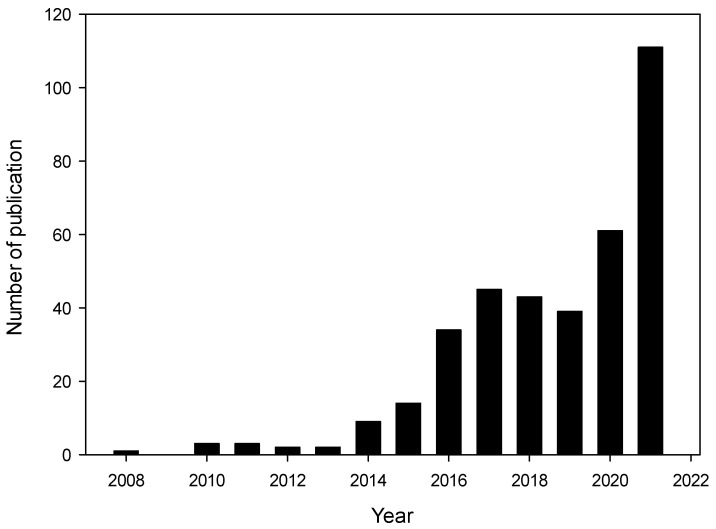
The distribution of publications related to ‘nanoemulsion as edible coating for fruits and vegetables’ (2005–2021): ScienceDirect databases. Data for 2021 is as of September 2021.

Figure 1 shows a survey of published scientific manuscripts on nanoemulsions as edible coatings for fruits and vegetables. The number of studies on the topic has increased considerably over the past few years, demonstrating the scientific community’s increased interest in the topic. However, studies concerning in vivo biological efficiencies are limited [14] and applications on fruits and vegetables are even fewer. Thus, more research is essential to determine this technology’s potential for future application on a commercial scale. In this context, the objective of this review is to update the available information on the use of nanoemulsions as coatings for preserving fresh fruits and vegetables.

## 2. Edible Coatings—An Overview

The first reports of the use of coatings on fruits appeared in the 12th century in China, where wax was applied to citrus (lemons and oranges) to reduce mass loss and preserve the fruit [15]. However, it was only in 1922 that the commercial scale application of waxes began in order to increase postharvest conservation of fruits and vegetables, thus reducing postharvest losses [16].

Currently, edible coatings are used as a strategy to increase the shelf life and postharvest quality of many fresh fruits and vegetables during storage [17,18]. Edible coatings are defined as thin layers applied on the fruit surface, forming clear films produced from food-grade materials and adding to, or as a substitute for, the waxes naturally present on the fruit surface. As these films become part of the food and are consumed as such (for fruits where the peel is consumed), the materials used in their composition must be GRAS (Generally Recognized as Safe), that is, be non-toxic and safe for food [19]. 

**Figure 2:**
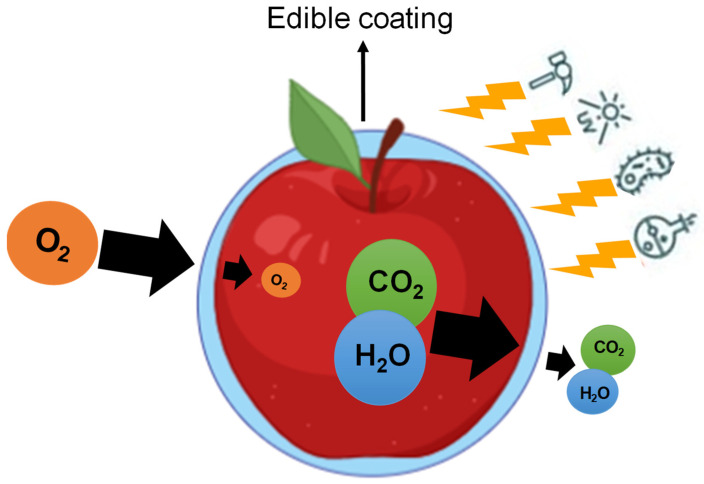
Main functions of edible coatings on fruits and vegetables.

Edible coatings are formulated from various biopolymers such as polysaccharide, lipid, and protein compounds, or by combining materials resulting in improved properties (Table 1). They act as an obstacle to water vapor, gases, and solutes [20] as shown in Figure 2.

The mechanism of action for coatings on fruit is similar to packaging with a modified atmosphere; the coating produces a physical barrier that modifies gas exchange between the interior of the fruit and the surrounding atmosphere, increasing the concentration of CO_2_ and decreasing O_2_ [30]. This environment can effectively decrease respiration rate, conserve stored energy, delay microbial growth, and therefore, extend the useful life of the fruit [31]. The coating efficiency depends on the coating thickness formed on the fruit surface, since there is a negative correlation between thickness and coating permeability [32]. Another important point is related to low permeability coatings, based on resins such as shellac, for example, which can restrict gas exchange almost entirely, leading to the accumulation of CO_2_ within the fruit, and the production of compounds resulting from the fermentation process that can cause off-flavor, such as acetaldehyde and ethanol, thus affecting fruit quality [18,33].

In addition to maintaining quality and postharvest conservation of fruits and vegetables, the coating materials can also act as carriers of compounds such as food coloring, flavoring, antimicrobials, antioxidants, antagonistic microorganisms, among others [34,35]. In this sense, several natural bioactive compounds have been incorporated into edible coating materials such as essential oils [36,37,38], plant extracts [39,40], vitamins [34], antagonistic microorganisms [41,42], and antibrowning or firming agents in fresh cut fruit. [43,44].

## 3. Methods to Apply Edible Coatings

The effectiveness of coatings in preserving fresh fruits and vegetables is influenced by the application method, which will be chosen according to the nature of the food to be coated, the surface attributes, the rheological properties of the solution, and the main purpose of the coating [45]. The adhesion of coatings to food surfaces is essential for performance of their intended function [16,46]. Wettability is used to quantify the interfacial interaction that occurs between the food surface and the coating. This variable must be taken into account when assessing the performance of the coating solution on the food surface [31].

**Figure 3:**
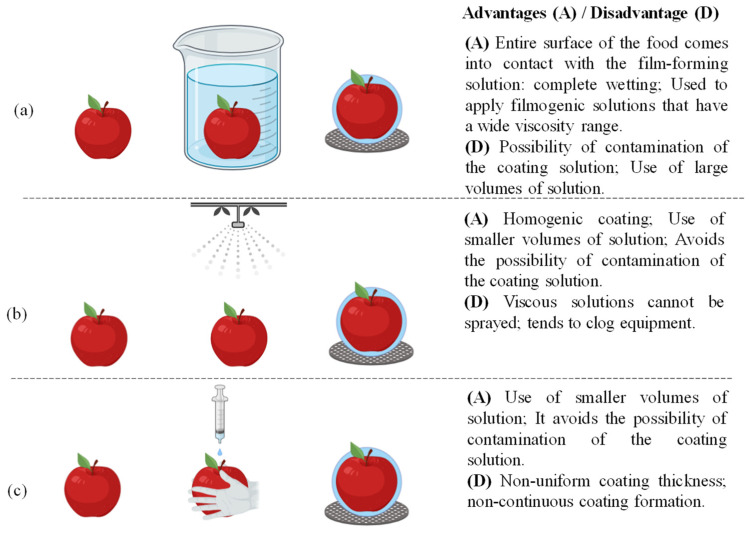
Dipping (**a**), spraying (**b**), and (**c**) hand coating techniques to apply edible coatings.

Dipping (Figure 3a), spraying (Figure 3b), and hand coating (Figure 3c) techniques are the most common methods for applying edible coatings to fresh fruits and vegetables. Other techniques such as fluidized bed and foaming are also available; however, these techniques are rarely used on commercial and laboratory scales [45].

On a laboratory scale, immersion is one of the main methods used for coating fruits due to its simplicity, without dependence on equipment, and uniformity of film obtained. In this method, the entire surface of the food is submerged in the film-forming solution at a constant speed, allowing full surface coverage, ensuring complete surface wetting [47]. After application, the excess solution is drained to eliminate the overload of film-forming solution on the fruit surface [48]. Finally, the food is dried with the excess solvent and liquid being evaporated to leave the film in contact with the food surface. Drying can take place at room temperature or using a heated air tunnel after draining the solution. This technique allows the application of coating solutions with a wide viscosity range [46]. A negative point of this technique is the possibility of cross-contamination from fruit to fruit during the immersion process due to the accumulation of residues and microbial organisms [45].

To avoid this problem, products that will be coated must be properly cleaned and sanitized, and the coating solution replaced frequently [15]. According to Raghav et al. [16], in general, fruits and vegetables are immersed for 5–30 s in the coating solution.

In turn, the spraying technique, most popular in packing houses, provides a homogeneous and attractive coating. In addition, it avoids the possibility of contaminating the coating solution [49]. This process increases the liquid surface through the formation of drops and distributes them over the food surface [45]. During spray application, the fruit or vegetable is placed on a plate or rotating rollers at a coordinated speed, under dispersing nozzles activated manually or automatically. This procedure is repeated until the desirable coating thickness is achieved. A drawback of this technique is that viscous solutions cannot be sprayed as they clog the equipment [50].

Another method to apply a filmogenic solution is by gloved hands to the fruit surface. Fruits can be coated by spreading a uniform amount of coating solution by hand while wearing latex gloves. It is appropriate on a laboratory scale to avoid solution contaminations and to minimize waste of experimental coating solutions during screenings. However, a negative aspect consists of the non-homogeneous film thickness formed on the entire fruit surface [18,35].

## 4. Nanomaterials in Edible Coatings

In recent years, nanotechnology has been used as an important tool to increase the storage period for food products. The application of nanoscale particles provides different and improved properties compared to particles with larger size. Related to foods, nanotechnology has a wide spectrum of uses in films and coatings due to the improved features they impart [51].

**Figure 4:**
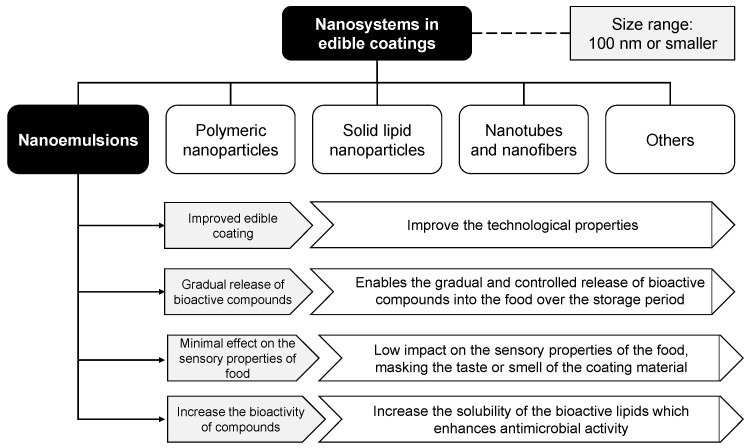
Nanomaterials in edible coatings.

Figure 4 shows the advances in the development of nanosystems incorporated with food-grade ingredients, which makes it feasible to explore functional modifications in food coating materials that include nanoemulsions, polymeric nanoparticles, nanostructured lipid transporters, nanotubes, nanocrystals, nanofibers, and others [52]. Nanosystems, when incorporated into matrices based on hydrocolloids (proteins or carbohydrates), give rise to nanocomposites, which are the combination of two or more materials, one of which is on a nanoscale, in order to improve coating properties [52,53].

The main changes due to use of nanosystems in nanocomposite coatings refer to the water barrier, optical and microstructural mechanical properties, and the antimicrobial and antioxidant effects. Nanoparticles in coatings potentiate these activities when antimicrobial or antioxidant compounds are incorporated in the coating, by enabling their gradual and controlled release over the period of fruit storage, sometimes under different storage conditions, hence improving bioavailability of these compounds over time [52,54]. The improvements in these properties are important to guarantee food quality maintenance as well as to reduce the development of decay microorganisms (bacteria, filamentous fungi, and yeasts) and action of free radicals that deteriorate food and reduce shelf life [55]. Another advantage of adding active agents to nanosystems is that a smaller proportion of these substances is necessary to obtain good activity; therefore, the use of these compounds in low concentrations does not negatively affect food sensory properties [12].

## 5. Fundamentals of Nanoemulsions 

**Figure 5:**
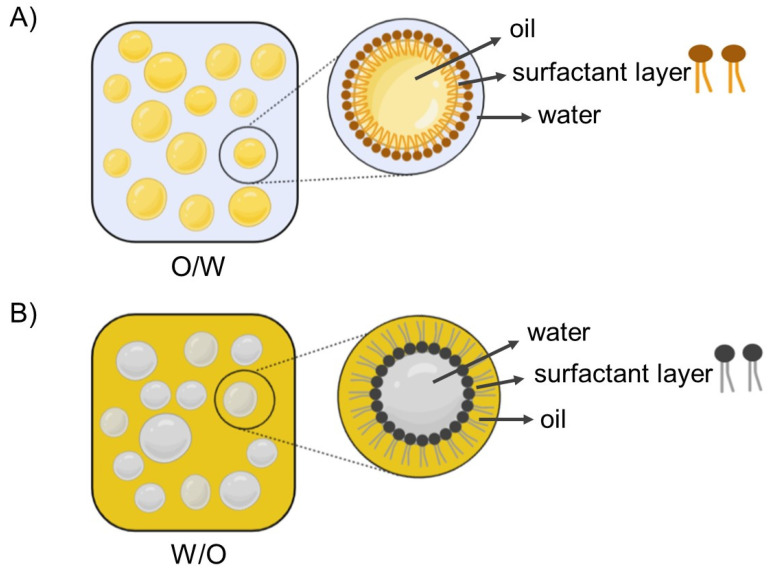
Schematic representation of (**A**) *oil-in-water* (*O/W*) and (**B**) *water-in-oil* (*W/O*) emulsions, representing micelle structure dispersed in continuous phase for each system.

Emulsions are generally made of two immiscible liquids, commonly oil and water, forming a relatively stable mixture. Generally, emulsions are systems that contain a dispersed and continuous phase and can be classified according to the three-dimensional organization of the oil and water phases. Oil-droplets dispersed within an aqueous phase is named *oil-in-water* (*O/W*) emulsion, whereas water-droplets dispersed in the oil phase is classified as *water-in-oil* (*W/O*) emulsion, and they are the most common emulsions [14,56,57]. Figure 5 shows the schematically structures of *O/W* (Figure 5A) and *W/O* (Figure 5B) emulsions, emphasizing the micelle structures dispersed in the continuous phase. 

Emulsions are classified into three main classes according to thermodynamic stability, stable mechanisms, and physical properties: macroemulsion or conventional emulsion, nanoemulsion, and microemulsion. Conventional and nanoemulsions are thermodynamically unstable, while the microemulsion is stable. The droplet mean radius for conventional emulsions are bigger, which distinguishes them from nanoemulsions with a radius of less than 100 nm [11,57,58]. The droplet size in nanoemulsions is a key-point that influences their capability to improve the bioavailability of added hydrophobic substances, such as carotenoids [58], and increase antimicrobial essential oil properties [59] or oil compounds [60]. The nanoemulsion classes will be further discussed in this article, with the focus of nanoemulsions as edible nanocoatings.

### 5.1. Nanoemulsions and Production Methods

The small size of particles in nanoemulsions allows potential advantages over conventional emulsions, such as greater stability concerning particle aggregation and gravitational separation, in addition to high optical transparency, modification of the physical properties of the coating, and increased bioavailability of bioactive-loaded lipid droplets [57]. Free nanoemulsion-based delivery systems increased the bioaccessibility of vitamins (D) and carotenoids (β-carotene and curcumin) [58,61]; however, studies have demonstrated that bioactive-loaded nanoemulsions prepared with a biopolymer mixture can be trapped in the matrices and decrease bioaccessibility. 

Nanoemulsions need energy for their formation, which is provided by mechanical equipment or physical and chemical properties of the system. Procedures using mechanical energy are called high energy methods and use microfluidizers, high-pressure homogenizers, and ultrasonic homogenizers. The methods that employ the system’s physical and chemical properties are categorized as low energy, such as spontaneous emulsification, phase inversion temperature, and emulsion inversion methods [54,57].

When high-energy methods are employed, the surfactants help break oil-droplets inside the homogenizer by decreasing interfacial tension, thus promoting smaller droplets and preventing droplet aggregation. A high shear rate is necessary to break the droplet to form nano-droplets, and is generally achieved by high-pressure homogenizers, as the use of high energy generates forces that can break the droplets in the dispersed phase [56,57]. Those methods are well established in the food industry and can be adapted for nanoemulsion production. On the other hand, for low energy methods, surfactants promote small droplet spontaneous formation due to their ability to generate extremely low interfacial tensions under specific conditions. Therefore, the surfactants utilized are extremely important because the emulsion pH stability, ionic strength, heating, cooling, and storage are mainly determined by the amphiphilic molecule chosen [56,57].

**Figure 6:**
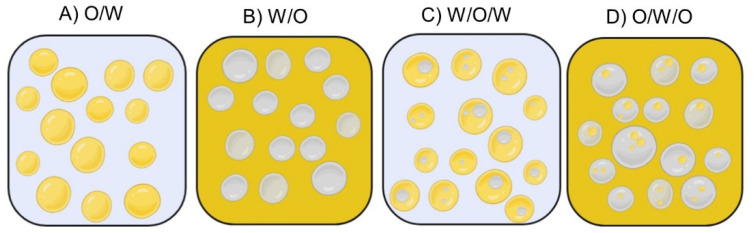
Representation of most common emulsion (**A**) *oil-in-water* (*O/W*) and (**B**) *water-in-oil* (*W/O*), and multiple emulsions (**C**) *water-in-oil-in-water* (*W/O/W*) and (**D**) *oil-in-water-in-oil* (*O/W/O*).

The amphiphilic material, such as surfactants, phospholipids, proteins, and polysaccharides, reduces the interfacial tension and maintains droplet stability. Emulsions (*O/W* or *W/O*) (Figure 6A,B) are the most stable systems; however in unusual regimes, multiple emulsions such as *W/O/W* and *O/W/O* (Figure 6C,D) may be formed and are usually extremely unstable to coalescence [14,54,56]. Most fruits and vegetables contain a high-water volume; therefore, among emulsions the *O/W* type (Figure 6A) is the most explored for food systems due to the possibility of loading the oil-droplets with lipophilic key-compounds surrounded by water [14,54].

### 5.2. Surfactants

Surfactants can be classified according to their electrical characteristics as ionic, non-ionic, and zwitterionic. Most foods surfactants are ionic, such as esterified monoglycerides, which are mainly negatively charged and can form nanoemulsions using low or high energy. Non-ionic surfactants also can be used for both methods and have low toxicity and irritability, including compounds such as Tween^®^ (condensate of sorbitol fatty acid esters and ethylene oxide) and Span^®^ (a family of fatty acids sorbitan). On the other hand, zwitterionic surfactants contain two or more ionizable groups with opposite charges, and consequently, they can have a negative, positive, or neutral charge depending on the pH solution. For example, this group includes lecithin, a phospholipid widely used in foods [57,62]. 

One of the main aspects of an emulsion formulation is the choice of surfactant. The hydrophilic–lipophilic Balance (HLB) system was developed, which represents the balance of the size and strength of the polar and non-polar groups [62]. It demonstrates molecule properties as amphiphilic compounds using a numerical scale, assigning higher HLB values as the substance is more hydrophilic [62]. However, the HLB system only considers the properties of the surfactant itself. For this reason, the hydrophilic–lipophilic deviation (HLD) system is another approach to the behavior exhibited by surfactant–oil–water and usually more suitable in formulations [57,63]. In addition, proteins, polymers with amphiphilic properties, and combinations of polymers and surfactants can act as emulsifiers [64]. 

Studies have demonstrated the importance of modulating nanoemulsions composition and structure to achieve higher digestion and absorption in the gastrointestinal tract and to efficiently deliver compounds such as vitamins and nutraceuticals [54,58,65,66]. Therefore, the choice of emulsifier is of extreme importance since it can improve carotenoids bio-accessibility, for example. In a study performed by Yao et al. (2019) [65], the authors demonstrated the relationship between carotenoids bio-accessibility from spinach and co-ingesting with excipient nanoemulsions: nanoemulsions containing different ratios of medium or long-chain triglycerides in the oil phase composition decreased β-carotene bioaccessibility when the ratio of medium-chain triglycerides was increased. The findings were credited to the formed micelle’s ability to hold the carotenoids in their hydrophobic domains.

## 6. Plant-Based Nanoemulsions as Edible Coatings on Fruits and Vegetables Postharvest 

### 6.1. Coatings Based on Essential Oil Nanoemulsions

One of the main features can be in the form of antimicrobial nanoemulsions, for example, nanoemulsions based on plant essential oils, which are associated with biopolymers such as alginate, chitosan, and starch, among others. It has been shown that when essential oils are encapsulated in nanoemulsions, they have less impact on the sensory properties of the food, masking the taste or smell of the core material (coating), yet providing better biological activity of essential oils due to the increase in the surface area [67]. In this way, it is possible to use low doses of bioactive material, increasing the transport of active ingredients through biological membranes, thus intensifying the bioavailability of bioactive compounds, in addition to less interaction with other components of the food matrix. Other advantages are the low mass transport of compounds into and out of the coating, less impact on optical, barrier, and microstructural properties and greater coating stability [68,69].

Essential oils have received special attention as active ingredients applicable in food coatings, due to their potent antimicrobial and antioxidant activities [70]. Essential oils are volatile aromatic substances of low molecular weight (for example, phenolic compounds, such as monoterpenes, flavonoids, and phenolic acids) produced by plants (for example, cinnamon, thyme, lavender, ginger, palmarosa, lemongrass, mint, citrus fruits, and fennel) or their isolated components (for example, eugenol, geraniol, menthol, limonene, carvacrol, and linalool) that can reduce microbial growth in food, and have been studied as natural antimicrobials in food for decades [71]. However, their volatile nature, low water solubility, and strong aroma limit their applications in foods. In this sense, using nanotechnological approaches is a promising strategy to enable the application of essential oils as natural antimicrobials in foods, overcoming their limitations and increasing their antimicrobial activity [52]. 

Table 2 presents the main types of nanoemulsions as edible coatings classified by matrix type and their impact on fruit and vegetable shelf life. 

Edible coatings based on nanoemulsions of essential oils have been studied as an alternative to prolong fresh fruit and vegetable shelf life. For example, a coating based on the nanoemulsion of lemon essential oil and chitosan increased the shelf life of arugula leaves by 7 days compared to a coating of chitosan or lemon oil alone [72]. Likewise, coatings based on modified chitosan and carvacrol nanoemulsions completely inhibited the growth of *Escherichia coli* on fresh green beans during the 11-day period under refrigeration [73]. Gundewadi et al. [74] also reported that the nanoemulsification of basil essential oil in an alginate coating was more effective than its respective microemulsion and presented better coating stability. In addition, when applied to okra fruits, nanoemulsion was more efficient in preserving texture, color, and sensory characteristics compared to control fruits. The essential oil of nanoemulsified basil showed greater antifungal activity against fungal pathogens than microemulsions. Chu et al. [75] developed a pullulan coating with a cinnamon essential oil nanoemulsion for strawberry storage. The nanoemulsion-based coating was more effective than other coatings in reducing loss of mass, firmness, total soluble solids, acidity, and controlling the growth of fungi and bacteria during fruit storage. 

In another study, Prakash, Baskaran, and Vadivel [60], evaluated the effect of a coating based on sodium alginate and citral nanoemulsion on the quality of fresh cut pineapples. Coatings based on nanoemulsions were effective at reducing microbial growth during storage. In addition, at a concentration of 0.2% of citral nanoemulsion, the coating reduced the presence of *Salmonella enterica* and *Listeria monocytogenes* after artificial inoculation [60]. The coating based on nanoemulsions of lemongrass essential oil, Tween^®^ 80 and alginate was more effective at preserving the characteristics of minimally processed Fuji apples than their respective conventional emulsions. The nanoemulsion coating inhibited the growth of artificially inoculated *E. coli* on fruits faster than conventional emulsions [59].

### 6.2. Coatings Based on Plant-Based Wax Nanoemulsions

Commercial coatings based on approved waxes must meet state/national fruit and vegetable additive regulations and be considered safe for consumption. However, to improve the characteristics of wax-based coatings, they are combined with synthetic chemicals to prevent microbiological deterioration and to ensure homogeneous stability of the coating during product storage. Commercial coatings are typically formulated using oxidized polyethylene wax (a by-product of the petroleum industry), carnauba wax (from the leaves of the carnauba palm, *Copernicia cerifera*), candelilla wax (from the candelilla shrub, *Euphorbia cerifera*), and shellac (from the insect bug *Kerria lacca*) as matrices, combined with water and other agents such as oleic acid, morpholine, ammonia, polydimethylsiloxane antifoam, and others [81]. 

The compounds combined with waxes used as emulsifying, moisturizing, and antimicrobial agents in commercial coatings, are mostly synthetic chemical products and could be a concern for human health [82]. As an example, morpholine is a base acting as a counterion to facilitate fatty acids emulsification in waxes. In the presence of nitrite/nitrate, morpholine can form N-nitrosomorpholine, a potent mutagen and carcinogen [83,84]. N-nitrosomorpholine was not found on coated fruit surface, but the possibility of its formation in the gut from reaction of morpholine with dietary nitrates was considered; it was found at concentrations less than the safe dose of 4.3 ng/kg body weight/day, not enough to raise concerns [85]. Ammonia could be used as a replacement for morpholine [86], but its highly volatile and irritant nature makes it less easy to use than morpholine. 

Consumers are increasingly concerned about the safety and quality of food, driving the demand for so-called “environmentally friendly coatings”, that is, coatings based on natural products of plant origin that do not present any harm to the consumer’s health if consumed. The use of waxes and compounds of animal origin has been limited by vegan and vegetarian consumers, consumers who are allergic to animal products (such as chitosan) and religious beliefs that do not encourage the consumption of animals [87]. Therefore, the demand for plant-based wax-based coatings is an important market for fresh fruits and vegetables, and nanotechnology is a promising tool to meet this demand by improving the properties of these coatings, especially wax-based ones, reducing the need of synthetic additives.

Nanotechnology has been successfully used to produce plant-based waxes nanoemulsions, such as carnauba wax [18] and candelilla wax [78] without the addition of morpholine. Wax-based nanoemulsions can have improved barrier properties due to the small size of the droplets, promoting greater homogeneity compared to conventional emulsions, greater transparency, improved physico-chemical properties (optical, mechanical, and barrier) and greater stability in comparison with conventional emulsions [18,54,78]. In addition, these nanoemulsions can be used for the development of nanocomposite coatings, in combination with hydrocolloid components (polysaccharides and proteins) in order to improve the water barrier properties of these compounds and minimize the impact of the incorporation of lipid compounds in the matrix hydrocolloids [29].

Lipid nanoemulsions made from plant-based waxes have shown greater effectiveness as edible coatings than conventional emulsions on fresh fruits and vegetables preservation (Table 2). The carnauba wax nanoemulsion coating showed less water loss, conferred gloss, and caused less ethanol production than shellac in coated ‘Nova’ mandarins (*Citrus reticulata*) and ‘Unique’ tangors (*C. sinensis*) [18]. In addition, the coating based on carnauba wax nanoemulsion exhibited less changes in the fruit internal atmosphere and volatile profile, and consequently, better flavor compared to the conventional carnauba wax emulsion and commercial shellac [18]. 

Lipid nanoemulsions produced from waxes, such as carnauba or candelilla, have been shown to be suitable vehicles for carrying bioactive compounds, such as plant extracts and essential oils [78,88]. They can improve the physical stability of the active substances, and improve the bioactivity of these compounds, and due to the prolonged and slow diffusion, they reduce the impact of these substances on the sensory properties of fruits and vegetables [88]. De Léon-Zapata et al. [78] developed candelilla wax nanoemulsions added with tarbush extract and evaluated its effect on the preservation of Fuji apples. The combination of extract and nanocoating reduced the size of the droplets and improved the zeta potential and optical properties of the coating. When applied to Fuji apples, the nanocoating effectively reduced physico-chemical and microbiological changes and delayed fruit senescence in comparison with the control treatment.

In another study, a nanoemulsion of carnauba wax combined with lemongrass essential oil nanoemulsion was applied to plums [76]. The coatings were able to inhibit the growth of *S. typhimurium* (*S. enterica*) and *E. coli* O157: H7 inoculated plums during storage, and did not significantly affect their taste and appearance (brightness). In addition, nanoemulsion coatings were effective at reducing weight loss, ethylene production and respiration rate. Fruit coated with nanoemulsions showed greater firmness and increase in phenolic compounds content during storage in comparison with uncoated fruits [76]. A similar result was observed in another study carried out by these authors with grape berries. The coating based on carnauba wax and lemongrass essential oil nanoemulsion inhibited the growth of *S. typhimurium* and *E. coli* O157: H7 inoculated fruit. Lemongrass in nanoemulsions did not affect berry taste and improved their brightness. Coatings based on nanoemulsions were also able to reduce weight loss and maintain firmness, phenolic compounds, and antioxidant activity in berries. The coatings demonstrated the potential to reduce microbiological contamination of grape berries by foodborne pathogens and prolong their shelf life. [77].

## 7. Trends in Materials Based on Nanoemulsions with Potential for Application in the Preservation of Fruits and Vegetables

New coating materials based on nanoemulsions with potential for application in fruits and vegetables have been developed in the last two years with the aim of contributing even more to the preservation of these products. One way to develop these functionalized materials is to combine composites with different properties to develop a functionalized coating. For example, de Oliveira Filho et al. [89] developed a functionalized coating combining arrowroot starch (biopolymeric matrix), carnauba wax nanoemulsion (to improve the water barrier properties of the coating), cellulose nanocrystals (to improve mechanical properties and stabilize the emulsion), and essential oils (to confer antimicrobial activity). The combination of compounds resulted in a coating material with excellent water barrier, mechanical, thermal, optical, microstructural, and antimicrobial properties against fungi that attack fruits during post-harvest. 

Another increasingly explored trend in the development of new coatings based on nanoemulsions with better stabilities is the use of solid particles to form Pikering nanoemulsions, that is, nanoemulsions stabilized with solid particles such as cellulose nanocrystals [90], starch nanocrystals [91], γ-Al2O3 nanoparticles [92], cyclodextrin [93], among others. 

Pickering nanoemulsions have excellent stability due to irreversible adsorption that occurs between solid particles at the oil–water interface due to the high adsorption energy [94]. Another characteristic of these nanoemulsions is the ability to release active ingredients encapsulated under specific conditions, such as pH and temperature [93]. Almasi, Azizi, and Amjadi [95] compared two coating materials based on pectin, one with marjoram essential oil encapsulated in a whey protein/inulin stabilized Pickering nanoemulsion, and the other with marjoram essential oil nanoemulsified with Tween 80. Coatings based on pectin with Pickering nanoemulsions presented mechanical and water barrier properties superior to those based on standard nanoemulsion. In another study, López-Monterrubio et al. [96] developed highly efficient β-carotene nanoemulsions stabilized by a complex formed by hydrolyzed whey protein and pectin. The nanoemulsions showed good stability during the 30-day storage period with low formation of clumps.

Deng et al. [80] developed coatings based on chitosan and Pickering nanoemulsion of oleic acid stabilized with cellulose nanocrystals and evaluated their effects on the postharvest conservation of green D’Anjou and Bartlett pears (*Pyrus communis* L.). The coating formulated with 5% cellulose nanocrystals showed strong adhesion to the fruit surface, showing greater gas barrier property compared to the commercial Semperfresh™ product, and presented a more homogeneous matrix, being effective in delaying ripening and increased the shelf life of pears during storage. 

Although the above new materials have been little studied in food systems, the results described in the literature are very encouraging.

## 8. Potential Toxicity, Limitations, and Regulatory Aspects of Nanoemulsions

Nanoemulsions, due to the nanometric size of the droplets, may partially remain intact during digestion, representing potential safety risks related to the compounds used for their production (such as surfactants). They can be of concern in metabolic or hormonal dysregulation due to their rapid absorption compared to conventional emulsions, their ability to increase the bioavailability of bioactive agents to a toxic level, and the possibility of increased absorption by epithelial cells which can cause changes in the functionality of the gastrointestinal tract [97]. However, as they have a high surface area, nanoemulsions can also be quickly digested by enzymes from the gastrointestinal tract, reducing the possible toxic effect that can occur due to their accumulation in organ cells [98].

In vitro studies were performed using cell cultures, usually models of normal cells such as fibroblasts, to investigate potential toxicity of nanoemulsions. Kaur et al. [99] reported that nanoemulsions based on tocopheryl polyethylene glycol succinate (TPGS), lemon oil, Tween-80, and water did not show toxicity in Hep G2 cells. In another study, Marchese et al. [100] observed that bergamot essential oil nanoemulsions showed cytotoxic activity against Caco 2 cells at high concentrations. A limitation of these studies is the fact that authors have not previously exposed the nanoemulsions in simulated conditions of the gastrointestinal tract before contact with the cells.

Knowledge about the potential toxicity of nanoemulsions in vivo is still limited and should be investigated [97]. The effect of nanoemulsions based on antimicrobial compounds, such as essential oils, on the gastrointestinal tract is also poorly reported in the literature. This effect must be carefully studied, as antimicrobial compounds can influence the intestinal microbiota or epithelial cells of the gastrointestinal tract.

In a recent study, Hort et al. [101] evaluated the toxicity of Miglyol and egg lecithin nanoemulsions using an in vivo model (male Wistar rats). The nanoemulsions were orally administered to rats for 21 days at lipid concentrations of 200, 400, or 800 mg/kg of body weight. The results of biochemical, hematological, oxidative stress, and genotoxicity parameters showed that nanoemulsions could be considered safe for oral administration, but high doses by the parenteral route could cause toxic effects.

The few studies suggest that nanoemulsions formulated with GRAS ingredients do not exhibit strong cytotoxic effects. The nanometer size of the droplets suggests that they are rapidly transformed into monoglycerides and free fatty acids in the small intestine, which are normal digestion products and should not have toxic effects [57].

As for regulatory aspects, essential oils and other antimicrobial agents are mainly regulated by the European Food Safety Authority (EFSA) in Europe and the Food and Drug Administration (FDA) in the United States [102]. However, for nanoemulsions there is no international authority that makes this regulation. The FDA addresses the regulation of nanotechnology products as guidance for industries. The European Council and Parliament have regulated food nanotechnology as new food products or food ingredients [103].

## 9. Conclusions and Future Perspectives 

The use of substances obtained from plant-based natural sources has emerged as a trend in the fresh fruit and vegetable market for coating applications. The application of these compounds on a nanoscale has advantages allowing a wider use in relation to particles on larger scales. Recent studies indicate that nanoemulsions play an important role in the development of a new generation of coatings with improved properties for the preservation of fresh fruits and vegetables. This emerging technology makes it possible to improve the physical stability and performance of active substances within an edible coating, bringing the possibility of increasing the quality and/or nutritional value of fruits and vegetables. Although the evidence published to date suggests that nanoemulsions applied as edible coatings can extend the life of different fruits and vegetables, there are other important aspects to explore before considering them on a commercial scale in future trends, such as the bioavailability of bioactive compounds incorporated in the nanoemulsions, potential toxicity and digestibility, for example. Most of the tested nanoemulsion coatings have antimicrobial properties; however, it can also be possible to produce and apply edible coatings with health-promoting substances.

## Figures and Tables

**Table 1:** Summary of diverse structural materials frequently used for edible coating.

Material	Main Matrices	Positive Points	Negative Points	References
Polysaccharide	Starch, chitosan, alginate, cellulose, and its derivatives, and pectin	Good gas and mechanical barrier properties	Poor moisture barrier due to hydrophilic nature	[21,22]
Lipid	Animal, vegetable waxes and resins, vegetable oil, and fatty acids	Good moisture barrier properties with a shiny appearance	Poor mechanical and gas barrier properties	[18,23,24]
Protein	Gelatin, casein, whey protein, zein, soy protein, myofibrillar protein, and quinoa protein	Good gas barrier properties without anaerobic conditions	Brittle and susceptible to cracking	[25]
Composite	Combination of polysaccharide and/or protein with lipids	Good moisture and gas barrier properties	Formation of non-homogeneous emulsion	[26,27,28,29]

**Table 2:** Main types nanoemulsions as edible coatings and their impact on fruit and vegetable shelf life.

Matrix	Bioactive Substance or Lipid Compound	Production Technique	Functionality	Fruit or Vegetable	Reference
Modified chitosan	Lemon, mandarin, oregano, or clove essential oils	High-pressure homogenization (HPH)	Increase the antimicrobial activity of the essential oil and improve the homogeneity and stability of the emulsion	Arugula leaf (*Eruca sativa*)	[72]
Chitosan	Carvacrol, bergamot, mandarin, and lemon essential oils	High-pressure homogenization	Increase the antimicrobial activity of essential oils	Green beans (*Phaseolus vulgaris*)	[73]
Sodium alginate	Basil essential oil	Ultrasound	Increase the antimicrobial activity of essential oil	Okra (*Abelmoschus esculentus*)	[74]
Pullulan	Cinnamon essential oil	Ultrasound	Improve the distribution of oil in the matrix and increase its antimicrobial activity	Strawberry (*Fragaria × ananassa*)	[75]
Carnauba wax	Lemongrass essential oil	Dynamic high pressure	Increase the antimicrobial activity of the essential oil and improve the homogeneity and stability of the emulsion	Plums (*Prunus salicina*)	[76]
Carnauba wax	Lemongrass essential oil	High shear probe and high-pressure dynamic processing (DHP)	Increase the antimicrobial activity of essential oil	Grape berry (*Vitis labruscana* Bailey)	[77]
Candelilla wax	Extract of tarbush	High-speed stirrer	Improved the wettability of the nanocoating on the Fuji apple surface	Fuji apple (*Malus domestica ‘Fuji*)	[78]
Quinoa protein/chitosan	Thymol	1200 rpm agitation	Increase the antimicrobial activity of the active compound and improve dispersion in the matrix	Strawberry (*Fragaria × ananassa*)	[79]
Sodium alginate	Lemongrass essential oil	Microfluidization	Improve the stability of the emulsion and increase the antimicrobial activity of the essential oil	Fresh-cut Fuji apples (*Malus domestica ‘Fuji*)	[59]
Hydroxypropyl methylcellulose	Carnauba wax nano-emulsion	High-pressure homogenization (HPH) and mechanical stirring	Reduce gas permeability and moisture loss	‘Redtainung’ Papaya (*Carica papaya*)	[28]
Sodium alginate	Citral	Ultrasound	Improve the dispersion of the active compound in the matrix and increase its antimicrobial activity	Fresh cut pineapples (*Ananas comosus*)	[60]
Carnauba wax	Oleic acid and Carnauba wax	High-pressure homogenization (HPH)	Improve optical properties, and emulsion stability	‘Nova’ mandarins (*Citrus reticulata*) and ‘Unique’ tangors (*C. reticulata C. sinensis*)	[18]
Chitosan	Cellulose nanocrystal and oleic acid	Ultra turrax homogenizer	Increase coating stability at high humidity, adhesion on fruit surface and delayed ripening of pears	Bartlett pears (*Pyrus communis*)	[80]
